# Differences in swallowing efficacy of disease modifying treatment between infants receiving pre-symptomatic and symptomatic administration

**DOI:** 10.1186/s13023-025-04049-9

**Published:** 2025-10-21

**Authors:** Katlyn Elizabeth McGrattan, Alicia Hofelich Mohr, Anna Miles, Jacqui Allen, Juliet Ochura, Kayla Hernandez, Katie Walsh, Vamshi Rao, Melanie Stevens, Heather McGhee, Keeley Nichols, Morgan Elaine Turksi, Abigail Spoden, Irena Wilson, Mackenzi Coker, Carmen Leon-Astudillo, Leann Schow Smith, John Brandsema, Hiba Farah, Julia Welc, Deborah Levy, Miranda Clements, Tina Duong, Sally Dunaway Young, Graham Schenck, Randal Richardson, Peter Karachunski, Ashley Brown, Allison Brown, Diana Castro, Basil T. Darras, Robert J. Graham

**Affiliations:** 1 University of Minnesota, Minneapolis, USA; 2 The University of Auckland, Auckland, New Zealand; 3 Boston Children’s Hospital, Boston, USA; 4 Lurie Children’s Hospital, Chicago, USA; 5 Ultragenyx, Novato, USA; 6 Nationwide Children’s Hospital, Columbus, USA; 7 Medical University of South Carolina, Charleston, USA; 8 Mayo Clinic, Rochester, USA; 9 Powell Gene Therapy Center, Department of Pediatrics, University of Florida College of Medicine, Gainesville, USA; 10 Primary Children’s Hospital, Salt Lake City, USA; 11 Children’s Hospital of Philadelphia, Philadelphia, USA; 12 Universidade Federal do Rio Grande do Sul, Hospital de Clínicas de Porto Alegre, Porto Alegre, Brazil; 13 Stanford Children’s Hospital, Palo Alto, USA; 14 School of Medicine, Stanford University, Stanford, USA; 15 Gillette Children’s Hospital, St Paul, USA; 16 Masonic Children’s Hospital, Minneapolis, USA; 17 Children’s Health System of Texas, Dallas, USA; 18 Neurology and Neuromuscular Care Center Texas, Denton, USA; 19 Boston Children’s Hospital, Harvard Medical School, Boston, USA

**Keywords:** Spinal muscular atrophy, Dysphagia, Swallow, Tube, Oral intake, Secretions, Aspiration

## Abstract

**Background:**

Spinal muscular atrophy causes progressive motor neuron degeneration that impedes an infant's ability to maintain full oral nutrition and manage secretions. Development of pharmaceuticals that halt neuromuscular degeneration have enabled survival and improvement in motor function, with infants who receive treatment before symptoms exhibiting better outcomes than those who receive treatment after symptom onset. Little is known about the impact of treatment timing on swallowing. We retrospectively evaluated swallowing biomechanics and function among infants who received a disease modifying treatment and a swallow study as part of routine clinical care at 13 international children's hospitals. Swallow studies were prospectively analyzed for measures of biomechanics using BabyVFSSImP© and Swallowtail, with chart reviews used to evaluate measures of function including oral intake status and secretion management. Data was reported with descriptive statistics, with differences in swallowing outcomes compared between infants who received pre-symptomatic and symptomatic treatment using non-parametric t-tests.

**Results:**

69 infants meeting eligibility criteria were included. The majority received treatment after symptom onset (N = 52, 75%) and had two copies of survival motor neuron 2 (SMN2) (pre-symptomatic N = 17, 100%; symptomatic N = 48, 92%). Median age of infants at the time of their last videofluoroscopic swallow study was 7.92 months (IQR 4.83). While profound impairments in swallowing biomechanics were rare among infants who received pre-symptomatic treatment, they were common among infants treated after symptom onset, with significantly worse (higher) scores in four BabyVFSSImP© domains (ts > 3.25, ps ≤ 0.01, δ > 0.42): Palatal-Pharyngeal Approximation, Airway Invasion/Laryngeal Closure, Aspiration, and Pharyngeal Transport and Clearance. Although all pre-symptomatic treated infants were managing secretions without suctioning and nearly all were consuming full age-appropriate nutrition (N = 15, 88%), similar to biomechanics, select pre-symptomatic treated infants did exhibit profound functional impairments.

**Conclusions:**

Infants who receive pre-symptomatic treatment for spinal muscular atrophy typically have good swallowing outcomes, without profound impairments in biomechanics, reliance on suctioning for secretion management, and reliance on alternative nutrition care. Pharyngeal biomechanical deficits are substantially more common among those infants that receive treatment after symptom onset, and likely are associated with subclinical neural degradation at the time treatment is administered.

**Supplementary Information:**

The online version contains supplementary material available at 10.1186/s13023-025-04049-9.

## Background

Spinal muscular atrophy (SMA) is a progressive neuromuscular disorder that leads to motor neuron degeneration and subsequent muscle weakness due to mutations in the survival motor neuron 1 (SMN1) gene. In its most severe form, this degeneration occurs rapidly within the first six months of life, and leaves infants with profound hypotonia [1–7]. Although hypotonia is widespread throughout skeletal muscles, the impact on the oropharyngeal musculature is of particular clinical relevance as it leads to profound impairments in the infant’s ability to efficiently suck to express milk, close the airway to prevent milk entry, and contract the pharynx to propel the milk into the esophagus [8–10]. The functional implications of these impairments are a reliance on external suctioning for management of saliva, alternative non-oral nutrition, and in many cases morbidity and mortality resulting from aspiration [8–12].

Development of pharmaceuticals capable of halting neuromuscular degeneration has transformed this previously inevitable downward trajectory to one of survival, and the obtainment of motor milestones previously unimaginable. The prognosis for the magnitude of these motor gains is closely linked with the timing of treatment administration. Pre-symptomatically treated infants exhibit better, often near normal motor outcomes [13], while those treated after symptom onset exhibit moderate developmental motor gains, with many continuing to encounter profound, though non-progressing, deficits [14–16].

Although the impact of disease modifying therapies on swallowing appears optimistic, the effects of treatment, and timing that it is provided, is not clearly understood. What has been reported is primarily limited to results from observational investigations reporting oral intake status. These results indicate the majority of infants treated prior to symptom onset maintain full oral feeds [17–19], while maintenance of full oral nutrition is far less common when treatment is initiated after symptom onset [18, 20, 21]. Even less is known about the integrity of swallowing biomechanics, though isolated case series suggest some swallowing impairments may persist [22]. The aim of this investigation was to elucidate differences in swallowing biomechanics and function between infants who receive treatment for SMA when they are pre-symptomatic compared to symptomatic.

## Methods

This is a retrospective multicenter investigation including 13 international children’s hospitals in the United States, New Zealand and Brazil. Select participants included in this investigation may have also been reported in previously published cross-sectional investigations by Hurst Davis et al. (2014) and Yuan et al. (2017) [23, 24] due to the shared sites and times for inclusion of participants. Seventeen participants who also had a videofluoroscopic swallow study (VFSS) prior to the administration of a disease modifying therapy were included in our previously published natural history analysis [8].

### Participant identification

Infants ≤ 12 months old who had genetically confirmed SMA and received a disease modifying treatment were the focus of the investigation. As our goal was to characterize the most severe phenotype, and historically 80% of untreated infants with two copies of SMN2 were characterized as an SMA Type 1 phenotype, this study included infants diagnosed with SMA if they were less than or equal to six months old at the time that symptoms presented, as well as infants treated prior to symptom onset with two copies of SMN2 [6]. Infants showing symptom onset at birth were excluded. Infants were included if they underwent a VFSS while on a disease modifying therapy as part of routine clinical care between January 2000-May 2025. In some medical centers, VFSS’ are performed solely if an infant has dysphagia symptoms or exhibited an impairment in the past, while at others it is done as part of a routine high-risk work-up regardless of symptoms. The sample, therefore, reflects these differences in referral pattern and is not a reflection of all children with SMA. Children were excluded from the investigation if they did not have a digital copy of their VFSS available for prospective analysis, or if their VFSS was completed at < 15 pulses per second. Although authors acknowledge that 30 pulses per second is optimal to maximize validity of exam results, this is not standard practice across medical centers. Fifteen pulses per second was therefore determined to provide sufficient validity to accomplish the study aims. Specifications of guidelines for VFSS execution in infants with SMA can be found in supplemental Table 1.

### Data collection

Eligible infants underwent medical record review by clinicians with expertise in neuromuscular disorders to gather information pertaining to demographics, SMA diagnostics, and medical management. Infants were categorized as being pre-symptomatic or symptomatic at the time of disease modifying therapy according to the Finkel and Benatar 2022 nosology stating symptomatic infants manifest clear symptoms of SMA recognizable to a trained neurologist [25]. Details pertaining to VFSS exams were collected by Speech Pathologists including age, reason for VFSS referral, and correlates of swallowing function including whether the child required external supports such as suctioning for secretion management (Y/N), respiratory supports, and their oral intake status as classified by the Children’s Eating and Drinking Activity Scale (CEDAS) [26]. CEDAS is a 6-point scale categorizing oral intake based on the presence and extent of tube use and the need for compensatory interventions like thickening [26]. Children’s Hospital of Philadelphia Infant Test of Neuromuscular Disorders (CHOP INTEND) scores taken closest to the last VFSS were recorded when available. CHOP INTEND is a disease-specific motor function assessment rating 16 areas of motor integrity on a 4-point scale for a best possible score of 64/64 representing highest motor function [27].

Digital files of VFSS exams from eligible infants were extracted from each site, de-identified, and shared with the core lab (University of Minnesota) for prospective analysis of swallowing. VFSS exams were conducted using barium contrast, with infants in varying positions ranging from side lying to upright, and in varying seating modalities ranging from the fluoroscopy bed to a tumbleform chair. Although all exams were conducted by fluoroscopically visualizing a sample of swallows, the timing of fluoroscopic evaluation and the number of swallows varied across patients and sites. This study was conducted in accordance with the principles of the Declaration of Helsinki and was approved by the University of Minnesota Institutional Review Board. The need for obtaining informed consent was waived because of the retrospective study design.

### Data analysis

De-identified VFSS exams were scored using BabyVFSSImP© and Swallowtail by raters who had achieved 80% reliability criteria and were blind to the infant’s background information. BabyVFSSImP© ranks integrity within 21 biomechanical components of oropharyngeal swallowing on an ordinal rank scale, with 0 representing the highest component integrity [28, 29]. Domain scores, summarizing performance into 5 biomechanical domains, can be calculated. In contrast to this categorical VFSS analysis approach, Swallowtail (Belldev Medical, Chicago, USA) is a method of VFSS analysis that uses a software platform to enable raters to make continuous measures pertaining to the timing and distance of swallow biomechanics according to the modified Leonard and Kendall approach [30]. Although this methodology allows for the assessment of 12 different biomechanics, for the purpose of this investigation, we strictly reported on pharyngeal constriction ratio and duration of pharyngoesophageal segment opening. These were selected as they were hypothesized to potentially offer greater granularity into swallowing biomechanics that may not be fully captured by BabyVFSSImP©, were able to be reliably rated on infants, and did not require systematic procedures regarding methods of videofluoroscopic capture not possible in this retrospective approach. Biomechanical integrity for the last available VFSS on file within the first year of life was reported. Swallowtail outcomes were summarized with averages, with BabyVFSSImP© summarized using overall impression scoring that assigns each biomechanical outcome a score based on the worst observed performance in an exam. Results are reported throughout the text pertaining to the proportion of infants exhibiting profound impairments within specific swallowing outcomes. Profound impairments in functional outcomes (oral intake and secretion management) were identified as a reliance on alternative nutrition and external secretion management supports respectively. While the lack of normative values for swallowing biomechanics in healthy, non-dysphagic children limits the delineation of normal performance from ‘mild’ score variants of physiologic processes, the interpretation of the most severe scale scores representing profound impairment leaves little room for debate. As such, profound impairment in BabyVFSSImP© biomechanics were delineated for the swallowing component that left no room for question as to their designation as representing severe impairment. A full listing of those score variants categorized as profound impairments can be found in supplemental Table 2. A cut off value of > 0.2 cm^2^ was used to define profound impairment in Swallowtail pharyngeal constriction ratio based on previous research which demonstrated 100 times greater risk of aspiration if pharyngeal constriction ratio was > 0.2 cm^2^ [31]. Inter-rater reliability was checked on 20% of exams, with discrepancies between raters resolved by consensus.

### Statistical analysis

Inter-rater reliability of BabyVFSSImP© ratings was calculated using weighted Cohen’s kappa coefficients, and reliability for Swallowtail was assessed using two-way consistency intraclass correlation coefficients (ICC). Characteristics of swallowing biomechanics and function for the last available VFSS for each infant were summarized using descriptive statistics including median (inter-quartile range) for domain scores and age, and proportions for individual components. Non-parametric t-tests were completed to test differences of BabyVFSSImP© domain scores between pre-symptomatic and symptomatic treatment groups, with effect sizes calculated using cliff's delta. The mean values across swallows were taken for the Swallowtail measures, and parametric t-tests were used to compare group differences in these values, with Cohen's d calculated for effect sizes. Chi-squared tests were used to test differences in impairment across specific BabyVFSSImP© component scores and functional outcomes, with Fisher's exact tests used in cases when expected counts were small and odds ratios (OR) calculated for effect sizes. Logistic regressions were used to assess the extent to which domain scores or Swallowtail measures predict functional impairment, with ANOVA used to compare model fit. All analyses were performed in R, with use of the nptest package [32] for all non-parametric t-test analyses, and an alpha level of 0.05. Given the rare nature of the disorder and required retrospective methodology, sample size was determined based on feasibility, using the largest number of exams accessible, rather than to achieve a specific power. A full export of the analysis can be found in the supplemental data file.

## Results

### Sample demographics and characteristics

Sixty-nine infants (49% female) meeting eligibility criteria were identified and included in the investigation. The majority received treatment after symptom onset (N = 52, 75%) and had two copies of SMN2 (pre-symptomatic N = 17, 100%; symptomatic N = 48, 92%) with others having three copies. All infants were treated with available disease modifying therapies (Risdiplam [Evrysdi®], Nusinersen [Spinraza®],Onasemnogene-abeparvovec [Zolgensma^®^]), with nearly half having received a combination of treatments (pre-symptomatic N = 10, 59%; symptomatic N = 28, 54%). Interestingly, ten (19%) of the infants who received treatment after symptom onset were identified as having SMA following SMA screening without symptoms triggering a referral and diagnosis. Upon initial neurology evaluation eight of those infants (80%) already exhibited clear symptoms of SMA (ex. Loss of reflexes) which were identified at an average age of 16 days. The other two did not exhibit symptoms at the time of their initial neurology consultation, though developed symptoms before their treatment was administered an average of 7 days later.

Infants treated pre-symptomatically received treatment at a younger age (median = 0.50, IQR 0.44 months), than those treated after symptom onset (2.81, IQR 3.56 months, t = 17.67, p < 0.001, δ = 0.89). Nine infants (13%) were born prematurely, with all but one being born in the late preterm period (median 36 weeks). Other comorbidities included laryngo or tracheomalacia (N = 3, 4%), biliary atresia (N = 1, 1%), craniosynostosis (N = 1, 1%), extralobar sequestration (N = 1, 1%), paraesophageal hernia (N = 1, 1%), and milk protein allergy (N = 1, 1%).

Median age of infants at the time of their last VFSS was 7.92 months (IQR 4.83), with infants who received pre-symptomatic treatment younger at the time of their last exam (6.8 IQR 5.77 months) than those who received treatment after symptoms (8.35, IQR 4.71; t = 2.96, p = 0.04, δ = 0.33). Although the majority (80%, N = 55) of infants underwent swallow studies due to clinical symptoms, 20% (N = 14) underwent the exam without symptoms as part of a routine, high-risk workup. Significantly more infants who received pre-symptomatic treatment underwent a routine, high-risk VFSS (59%, N = 10) than did those who received symptomatic treatment (8%, N = 4, p < 0.001, OR 0.06). Nearly all infants (86%, N = 59) were evaluated while consuming thin liquids, with other consistencies observed including mildly thick (43%, N = 30), moderately thick (22%, N = 15).

At the time of last VFSS, 50% (N = 26) of symptomatic treated infants were receiving noninvasive ventilation or mechanical ventilation via tracheostomy (22/26, 85% strictly nocturnal), while only one of those treated pre-symptomatic required these supports (strictly nocturnal) (χ^2^ = 9.43, p = 0.002, OR 17.28). CHOP INTEND scores were available for 66% of infants, with a median score of 46 [16] out of 64. Table 1 provides a full listing of infant demographics and clinical characteristics.

**Table 1:** Sample demographics and characteristics (N = 69)

	Pre-Symptomatic *N* = *17*	Symptomatic *N* = *52*
Female	8 (47%)	26 (50%)
Race		
Asian	1 (6%)	5 (10%)
Black or African American	1 (6%)	1 (2%)
Native Hawaiian or Pacific Islander	0 (0%)	1 (2%)
Native American/Alaskan Native	0 (0%)	1 (2%)
White	11 (65%)	34 (65%)
Unknown	4 (24%)	10 (19%)
Ethnicity		
Hispanic or Latino	1 (6%)	6 (12%)
Not Hispanic or Latino	9 (53%)	38 (73%)
Unknown	7 (41%)	8 (15%)
SMN2 Copy Number		
2	17 (100%)	48 (92%)
3	0 (0%)	4 (8%)
Initial Disease Modifying Treatment Age (months)*	0.50 (0.44) *0.00–0.85*	2.81 (3.56) *0.39–11.00*
Evrysdi® (Risdiplam)	2 (12%)	1 (2%)
Spinraza® (Nusinersen)	1 (6%)	20 (38%)
Zolgensma® (Onasemnogene Abeparvovec)	4 (24%)	3 (6%)
Combination	10 (59%)	28 (54%)
VFSS Indication: Clinical Symptoms*	7 (42%)	48 (92%)
Age at Last VFSS (months)*	6.80 (5.77) 0.26–12.00	8.35 (4.71) 0.92–12.88

Values reflect median (IQR) Min–Max, and N (proportion)

Proportions reflect the number of participants out of the sample with data available for the outcome of interest. They may not add to 100% due to rounding

^*^Indicates significant differences between groups

### Swallow biomechanics

Raters achieved scores corresponding to a Landis-Koch category of moderate or greater (kappa > 0.55) agreement in their reliability of analyzing all BabyVFSSImP© and ICCs of ≥ 0.72 for the Swallowtail components. While profound impairments in BabyVFSSImP© swallowing biomechanics were rare among infants who received pre-symptomatic treatment, they were common among infants treated after symptom onset. This was reflected in significantly worse (higher) scores in four BabyVFSSImP© domains (ts > 3.25, ps ≤ 0.01, δ > 0.42): Palatal-Pharyngeal Approximation, Airway Invasion/Laryngeal Closure, Aspiration, and Pharyngeal Transport and Clearance (Table 2). Specifications of the oropharyngeal swallowing biomechanics underlying these differences across treatment groups are outlined below, with a full listing of BabyVFSSImP© component scores provided in supplemental Table 2.

**Table 2:** Swallowing Biomechanics by Treatment Group (N = 69)

	Pre-Symptomatic *N* = 17*	Symptomatic *N* = *52*	P; δ or d
BabyVFSSImP©			
I: Pharyngeal Swallow Initiation *Possible Range: 0–18*	12 (3)	15 (6)	0.05; 0.3
II: Palatal-Pharyngeal Approximation *Possible Range: 0–5*	0 (0)	2 (4)	0.026; 0.41
III: Airway Invasion/Laryngeal Closure *Possible Range: 0–15*	9 (2)	11 (2)	< 0.001; 0.52
IV: Aspiration *Possible Range: 0–5*	0 (3)	3 (4)	0.005; 0.42
V: Pharyngeal Transport and Clearance *Possible Range: 0–19*	7 (3)	9 (5.3)	< 0.001; 0.63
SwallowTail			
Pharyngeal Constriction Ratio *Possible Range: 0–1*	0.03 (0.02)	0.15 (0.16)	< 0.001; 0.87
Pharyngoesophageal Segment Opening Duration *Possible Range: 0-No Maximum*	0.22 (0.06)	0.24 (0.09)	0.4 0.2

Median domain scores (Interquartile Range) and results of non-parametric t-tests (p and absolute value of cliff's delta). Lower scores represent better performance for all outcomes except pharyngoesophageal segment opening duration, with 0 being the best score, and the worst possible performance specified for each outcome respectively

^*^N = 20 for domains II and IV due to poor image quality for one infant

Bolus Extraction: Though the majority (76%, N = 13) of infants who received pre-symptomatic treatment promptly initiated sucking when presented with the nipple, all but one had to suck > 3 times to express sufficient bolus to swallow. Prompt initiation of sucking tended to be less common in infants treated after symptom onset (40%, N = 21), with 55% (N = 28) of these infants not initiating sucking at all (χ^2^(1) = 3.59, p = 0.06, OR 0.28).

Bolus Clearance: Infants who received pre-symptomatic treatment rarely exhibited profound impairments in the ability to clear the ingested liquid from their pharynx, with no infants exhibiting profound impairments in pharyngeal constriction ratio (PCR > 0.2cm^2^), tongue base retraction, or pharyngeal residue, and only one infant exhibiting profound reductions in soft palate elevation (6%, N = 1) and pharyngoesophageal segment opening (6%, N = 1). Clinical specifications of those pre-symptomatic infants with profound impairments are outlined in Table 3. This was in contrast to infants treated after symptom onset, for whom a significantly higher proportion of infants (23–43%) exhibited profound impairments in these processes (BabyVFSSImP©, χ^2^(1) > 4.45, p ≤ 0.03, OR > 9.21; SwallowTail pharyngeal constriction ratio, fisher's exact p = 0.03, OR = 0). These deficits in the propulsion of the bolus through the pharynx among infants treated after symptom onset were reflected in elevated Swallowtail pharyngeal constriction ratio values (symptomatic m = 0.15, sd = 0.16; pre-symptomatic m = 0.03, sd = 0.02; t (53.80) = 5.10, p < 0.001, d = 0.88). No differences were observed between groups in pharyngoesophageal segment opening duration (symptomatic m = 0.24, sd = 0.09; pre-symptomatic m = 0.22, sd = 0.06); t(40.04) = 0.84, p = 0.41, d = 0.20).

**Table 3:** Clinical Characteristics of Pre-Sympomatic Infants with Profound Swallowing Impairments

Participant	Detection method	Treatment type and age	Days after treatment that dysphagia symptoms reported	Details surrounding impairment
14	Newborn Screen	2 weeks Zolgensma®	11	Admitted with RSV 11 days post treatment requiring intubation. Tube feedings were initiated at this time and utilized at discharge. VFSS at 3 months limited as the infant did not initiate sucking, but limited presentations of thin liquid revealing absent epiglottic inversion and pharyngeal stripping wave, with minimal pharyngoesophageal segment opening. Full oral intake achieved at 8 months old
24	Newborn Screen	1 week Evrysdi® 2 weeks Zolgenzma®	11	2 days after Evrysdi® for bridging started taking steroids in preparation for Zolgensma®. Vomiting noted this date, though attributed to steroids. Zolgensma® administered at 2 weeks, with a notable drop in weight growth curve from 28% at birth to 11% at 11 days following initial Evrysdi® treatment. Continued on steroids until 2.5 months during which time emesis continued to increase, constipation, trouble latching to the nipple, and continued impairment in weight gain (0.5%). Tube placed 3.5 months old with improvement in weight gain, however vomiting continued to get worse despite normal upper endoscopy results, reflux management, and formula changes. VFSS done at 6 months was limited as infant would not latch, though observed swallows without deficits in bolus clearance or airway protection. Still using tube with daily emesis at 12 months

Airway Protection: Penetration occurred in almost all infants in both pre-symptomatic (95%, N = 16) and symptomatic (90%, N = 47) treatment groups (p = 1). Although aspiration occurred less frequently, it was still commonly observed; occurring more frequently in infants treated after symptom onset (71%, N = 36) than pre-symptomatic (35%. N = 6, χ^2^(1) = 5.31, p = 0.02, OR 4.4). Interestingly, among those infants who aspirated, 17% (N = 7) were not reported to be exhibiting feeding difficulties, with VFSS’ done as part of high-risk referral (pre-symptomatic 67%, N = 4; symptomatic 8%, N = 3).

### Swallow function

Although all pre-symptomatic treated infants were managing secretions without suctioning, and nearly all were consuming full age-appropriate nutrition (N = 15, 88%), similar to biomechanics, some pre-symptomatic treated infants did exhibit profound functional impairments. Clinical deficits among those pre-symptomatic treated infants who required alternative nutrition appeared 11 days following treatment, with the reason for tube provision ranging from swallowing deficits impeding safe oral nutrition to impairments in consuming sufficient oral nutrition to meet caloric requirements. Table 3 provides further specifications pertaining to the clinical conditions of those pre-symptomatic infants who exhibited profound impairments. Significantly more infants treated after symptom onset required suctioning for secretion management (38% vs 0%; fisher's p = 0.002, OR 0) and were not consuming age-appropriate oral nutrition (50% vs. 12%; χ^2^ (1) = 17.33, p < 0.001, OR 20.35) than those treated pre-symptomatic. Table 4 provides a full listing of CEDAS scores.

**Table 4:** Children’s Eating and Drinking Activity Scale (CEDAS)

	Pre-Symptomatic *N* = *17*	Symptomatic *N* = *52*
1: Tube use for all nutrition and hydration. Nothing by mouth. Non-nutritive sucking and/or mouthcare only	0 (0%)	10 (19%)
2: Tube use for all nutrition and hydration. Oral intake for experience and/or pleasure only	0 (0%)	7 (13%)
3: Tube use with consistent intake of food and/or drink. Oral intake partially meets nutrition and/or hydration needs	2 (12%)	9 (17%)
4: Total oral intake, requiring special preparation of food or drinks (IDDSI level 1–4) and/or food (IDDSI level 3–5 where not age appropriate) and/or supplements needed for nutritional support	0 (0%)	5 (10%)
5: Total oral intake, requiring special conditions (ex. equipment, pacing) or food modification as IDDSI level 6–7 (where not age appropriate) or food types/groups restricted by avoidance (where not age appropriate) but without need for supplements	0 (0%)	7 (13%)
6: Total oral intake. Age-appropriate food and drink with no restrictions	15 (88%)	14 (27%)

Swallow biomechanics were associated with swallowing function, with the odds of an infant requiring suctioning for secretion management increasing significantly with increases in BabyVFSSImP© domain V: pharyngeal transport and bolus clearance scores (β = 0.30, z = 2.76, p = 0.006) and Swallowtail pharyngeal constriction ratio (β = 3.76, z = 1.83, p = 0.047). Among those domain V components, pharyngeal stripping wave was particularly predictive of suctioning needs (fisher's exact p = 0.001, OR = 9.78). In contrast, the odds of an infant not achieving age-appropriate nutrition or hydration was significantly predicted by increases in domain I: lingual motion/swallow initiation (β = 0.26, z = 2.49, p = 0.013).

## Discussion

This investigation is the first to characterize swallowing outcomes after disease modifying treatment in a large sample of infants with SMA using validated swallowing outcomes. This fills a tremendous void for clinicians seeking to understand implications of timing of disease modifying therapy administration and counsel families regarding anticipated swallowing outcomes. The key findings from this investigation indicate (1) infants who receive disease modifying treatment after symptom onset may still experience profound swallowing impairments; (2) infants who receive pre-symptomatic treatment have significantly better, albeit not necessarily normal, swallowing outcomes; and (3) deficits in airway protection resulting in penetration and aspiration are common across both pre-symptomatic and symptomatic treated infants.

Our results revealed many of the infants treated after symptom onset still exhibited swallowing deficits, often profound in severity, in the swallowing biomechanics that facilitate secretion management and oral nutrition. Previous work examining swallowing outcomes in a small number of infants (≤ 10) treated after symptom onset (average treatment age 29 days-4mths) report 80–100% of infants exhibited impairment in sucking [20], bolus clearance, and airway protection [22, 33, 34], leading to the need for alternative nutrition in 14–100% of infants [18, 35–37]. Our findings are consistent with this work, with 88–92% of infants exhibiting some evidence of deviation in these pharyngeal biomechanics, and 49% of infants relying on alternative nutrition. Similar trends have been reported in gross motor integrity. For example, Finkel et al. (2021) reported 58–89% of infants treated after symptom onset exhibit persistent delays in head control, sitting unassisted, crawling, or walking [15].

A key consideration in interpreting these results is that although disease modifying treatments have demonstrated efficacy in halting future motor neuron death resulting from insufficient SMN protein levels, none are designed to replace those motor neurons that were already lost at the time of treatment provision. Our previous work elucidating the timing of swallowing degradation in an untreated, natural history sample of infants with SMA 1 found that by three months old many infants already exhibited profound impairments in the processes that facilitate airway protection (97%) and bolus clearance (37%) [8]. Given the average age of treatment in the current investigation was three months old, it is likely that motor neurons controlling swallowing were already impacted for many infants at the timing of treatment administration. Future prospective investigations examining how pre-treatment swallowing biomechanics predict post-treatment outcomes are necessary to develop better prognostic models.

This premise supports our second key finding that infants who receive pre-symptomatic treatment typically have very good swallowing outcomes. Eighty-eight percent of our sample was consuming full age-appropriate oral intake at their last study timepoint, with profound deficits in the clearance of the bolus from the pharynx limited to just one infant. These promising outcomes among infants receiving treatment by one month old, as well as our findings of select infants in the neonatal period going from pre-symptomatic to symptomatic in less than 2 weeks, underscores the importance of newborn screening to facilitate rapid treatment before damage has occurred.

Though these optimistic outcomes are encouraging, attention to the minority who had less favorable swallowing outcomes is of great importance to the field moving forward. Two (12%), pre-symptomatic infants required alternative nutrition, one of which had profound impairments in bolus clearance, and strikingly, 6 (36%) aspirated. These biomechanical and function deficits amidst early treatment may shed light on just how early motor neurons facilitating key bulbar functions are impacted, and potentially provide prognostic value. SMA has a wide spectrum of severities impacting timing of manifestation, and rate of disease progression [6]. It is now appreciated that there is a prodromal phase of disease manifestation before true symptomatic presentation. During this phase motor neuron loss is occurring, though clinical indices of degradation are subtle and not typically detectable without specialized neurologic and motor assessment [25] Recent work has demonstrated infants treated in this prodromal phase may have worse swallowing outcomes than those treated before [38]. Given the biomechanics that facilitate swallowing are internal, clinical signs of mild to moderate swallowing impairment are often missed [8]. This is especially true among infants who have not yet developed a cough reflex in response to events like aspiration [39]. It is plausible that more pre-symptomatic infants than currently appreciated have mild to moderate deficits in swallowing biomechanics, and underscores the importance of routine high-risk instrumental assessment. Swallowing processes that facilitate airway protection may be particularly suspectable to reduced neuromuscular integirity, as successful execution not only requires integrity within neuromotor control of the oropharyngeal musculature, but also within the respiratory system to coordinate the interchange between ventilation and respiratory cessation. Future prospective investigations systematically elucidating swallowing biomechanics in pre-symptomatic infants are needed to understand the prevalence of these deficits and develop care guidelines surrounding swallowing assessment and treatment in this population.

The retrospective nature of this investigation brings with it several limitations that must be considered in the interpretation of these results. While by virtue of inclusion criteria all infants underwent a swallow study as part of routine clinical care, the reasons for VFSS referral were variable across institutions and providers. For example, some providers strictly referred infants when symptoms of swallowing impairment presented, while others referred all infants diagnosed with SMA as part of a routine, high-risk referral regardless of dysphagia symptoms. Given this, reported rates of impairment should be interpreted with caution and not generalized to prevalence across all treated infants with SMA. Similarly, infants who received pre-symptomatic treatment were more likely to have their VFSS at a younger age as part of a high-risk VFSS referral. This is likely a reflection of inherent differences in what is a possible referral pattern between groups, as infants who receive treatment after symptom onset typically already have dysphagia symptoms that would result in a referral for a VFSS exam. Unfortunately, due to sample size limitations we were not able to control for this variable, and as such, readers should interpret these results with those differences in mind. It is also worth acknowledging that determination of pre-treatment symptomology may vary between providers with subtle differences in gross motor findings corresponding clinically significant swallowing deficits. Lastly, we strictly reported swallowing outcomes for the first year of life due to changes from bottle to cup drinking that occurs thereafter and impedes our evaluation of all outcome metrics over time. It is plausible that swallowing changes may continue well beyond the first year of life, with further investigations into these long-term outcomes warranted.

## Conclusions

Infants who receive pre-symptomatic treatment for SMA typically have overall good swallowing outcomes, without profound impairments in biomechanics, reliance on suctioning for secretion management, and reliance on alternative nutrition care. These deficits are substantially more common among those infants that receive treatment after symptom onset and may be associated with subclinical prodromal neural degradation at the time treatment is administered. Future prospective investigations that evaluate swallowing before and after treatment among pre-symptomatic infants are necessary to further understand the prevalence and significance of these impairments.

## Supplementary Information

Additional file 1.

## Data Availability

The datasets generated during and/or analyzed during the current study are not available per scope of IRB approval, however all analyzed data and code is available in GitHub: https://github.com/katlynmcgrattan/SMA-Pre-Post-Treatment/.
