# Supplementary material for: Differences in swallowing efficacy of disease modifying treatment between infants receiving pre-symptomatic and symptomatic administration
Source: Orphanet J Rare Dis. 2025 Oct 21;20:526. doi: 10.1186/s13023-025-04049-9 (PMC12539221; doi:10.1186/s13023-025-04049-9)
Supplement: Supplementary file 1 — Additional file 1. [file 13023_2025_4049_MOESM1_ESM.docx]

Supplemental Table 2: Guidelines for VFSS Execution in Newly Diagnosed Infants with SMA

| **Clinical Indication** | Routine, high-risk instrumental swallow assessments regardless of dysphagia symptoms should be conducted on all newly diagnosed infants with SMA when clinically available. This is due to the fact that available data indicates degradation of bulbar motor neurons often occurs very early on in disease progression without overt clinical symptoms, and integrity at the time of treatment may offer valuable information pertaining to future clinical management and bulbar prognosis. |
| --- | --- |
| **Fluoroscopic Pulse Rate** | Running fluoroscopy at 30 pulses per second (pps) (continuous) is optimal to maximize diagnostic yield and should be utilized whenever possible. In centers where equipment limitations prohibit the use of 30 pps pulse rate clinicians should work with their radiology partners to execute the exam at the next highest available pulse rate. |
| **Field of View** | To maximize image quality and reduce radiation exposure exams should be collimated to include the lips anteriorly, cervical spine posteriorly, nasal cavity superiorly, and upper esophageal segment inferiorly. |
| **Contrast** | Varibar® barium is the only standardized contrast designed for VFSS use. As such, it should be used at sites where it is approved. Sites that do not have approval should use their available barium contrast, mixing it to all thickness levels according to IDDSI criteria. |
| **Fluoroscopic Protocol** | Whenever feasible by infant compliance exams should be completed according to the Charleston protocol which includes fluoroscopic visualization of five swallows at (min:sec) 00:00, 00:30, 01:30, and 02:30 while the infant consumes thin barium contrast without attempts to remove the nipple to allow for assessment of fatigueability. |

Supplemental Table 1: BabyVFSSImP© Component Scores at Last VFSS

| **BabyVFSSImP**© **Component Score** | **Pre-Symptomatic**  *N=17* | **Symptomatic**  *N=52** |
| --- | --- | --- |
| **Domain 1: Lingual Motion/Pharyngeal Swallow Initiation** | | |
| **Initiation of Nutritive Sucking** | | |
| (0) Prompt | 12 (71%) | 21 (40%) |
| 1. Delayed | 1 (6%) | 3 (6%) |
| 1. No Initiation | 4 (24%) | 28 (54%) |
| **Number of Sucks to Form Bolus** | | |
| 1. Sucking 1 time | 0 (0%) | 0 (0%) |
| 1. Sucking 2 times | 0 (0%) | 2 (4%) |
| 1. Sucking 3 times | 1 (6%) | 2 (4%) |
| 1. Sucking 4 times | 2 (12%) | 7 (13%) |
| 1. Sucking 5 times | 5 (29%) | 5 (10%) |
| 1. Sucking 6 times | 5 (29%) | 7 (13%) |
| 1. Sucking without extraction or no sucking established. | 4 (24%) | 29 (56%) |
| **Nutritive Sucking Rhythmicity** | | |
| (0) Organized | 1 (6%) | 5 (10%) |
| 1. Intermittent | 11 (65%) | 20 (38%) |
| 1. Disorganized | 6 (29%) | 27 (52%) |
| **Suck/Swallow Bolus Control** | | |
| (0) Cohesive Bolus Contained in the oral cavity | 0 (0%) | 0 (0%) |
| 1. Diffuse bolus contained in oral cavity | 0 (0%) | 0 (0%) |
| 1. Contrast escape to the pharynx | 17 (100%) | 52 (100%) |
| **Bolus Location at Initiation of Pharyngeal Swallow** | | |
| (0) Above or at valleculae | 1 (6%) | 1 (2%) |
| 1. Between valleculae and pyriforms | 0 (0%) | 0 (0%) |
| 1. In pyriforms | 16 (94%) | 49 (94%) |
| 1. No initiation | 0 (0%) | 2 (4%) |
| **Timing of Initiation of Pharyngeal Swallow** | | |
| (0) Immediate or ≤ 1 second | 6 (35%) | 15 (29%) |
| 1. >1 but ≤ 2 seconds | 5 (29%) | 9 (17%) |
| 1. >2 seconds | 6 (35%) | 28 (54%) |
| **Domain II: Palatal-Pharyngeal Approximation** | | |
| **Palatal-Pharyngeal Approximation/Palatal Integrity** | | |
| (0) No contrast between soft palate (SP) and posterior pharyngeal wall (PPW) | 13 (76%) | 23 (44%) |
| 1. Trace column of contrast | 3 (18%) | 7 (13%) |
| 1. Narrow column of contrast^P^ | 1 (6%) | 18 (35%) |
| 1. Wide column of contrast^P^ | 0 (0%) | 4 (8%) |
| **Location of Bolus at Time of Palatal-Pharyngeal Approximation** | | |
| (0) No contrast entry beyond the oropharynx | 13 (76%) | 22 (42%) |
| 1. Contrast entry to nasopharynx | 3 (18%) | 10 (19%) |
| 1. Contrast entry to the nasal cavity^P^ | 1 (6%) | 20 (38%) |
| **Domain III: Airway Invasion/Laryngeal Closure** | | |
| **Early Laryngeal Vestibular Closure** | | |
| (0) Complete; no air/contrast in vestibule | 1 (6%) | 5 (10%) |
| 1. Trace column of air/contrast in vestibule | 4 (24%) | 3 (6%) |
| 1. Narrow column of air/contrast in the vestibule | 11 (65%) | 34 (71%) |
| 1. Wide column of air/contrast in the laryngeal vestibule | 1 (6%) | 7 (14%) |
| **Late Laryngeal Vestibular Closure** | | |
| (0) Complete; no air/contrast in vestibule | 7 (41%) | 6 (12%) |
| 1. Trace column of air/contrast in vestibule | 10 (49%) | 22 (43%) |
| 1. Narrow column of air/contrast in the vestibule^P^ | 0 (0%) | 21 (41%) |
| 1. Wide column of air/contrast in the laryngeal vestibule^P^ | 0 (0%) | 2 (4%) |
| **Timing of Airway Entry** | | |
| (0) None | 1 (6%) | 4 (8%) |
| 1. Pre-Swallow | 1 (6%) | 2 (4%) |
| 1. During Swallow | 10 (59%) | 11 (22%) |
| 1. Post-Swallow | 1 (6%) | 0 (0%) |
| 1. Any combination of 2 or more of the above | 4 (24%) | 34 (67%) |
| **Amount of Penetration** | | |
| (0) None | 1 (6%) | 4 (8%) |
| 1. Trace | 4 (24%) | 4 (8%) |
| 1. More than trace | 12 (71%) | 43 (84%) |
| **Frequency of Penetration** | | |
| (0) None | 1 (6%) | 4 (8%) |
| 1. 1 swallow | 2 (12%) | 4 (8%) |
| 1. Intermittent | 9 (53%) | 28 (55%) |
| 1. Repeated | 5 (29%) | 15 (29%) |
| **Domain IV: Aspiration** | | |
| **Amount of Aspiration** | | |
| (0) None | 11 (65%) | 15 (29%) |
| (2) Trace | 4 (24%) | 8 (16%) |
| 1. More than trace | 2 (12%) | 28 (55%) |
| **Frequency of Aspiration** | | |
| (0) None | 11 (65%) | 15 (29%) |
| 1. 1 swallow | 1 (6%) | 10 (20%) |
| 1. Intermittent | 5 (29%) | 25 (49%) |
| 1. Repeated | 0 (0%) | 1 (2%) |
| **Domain V: Pharyngeal Transport and Clearance** | | |
| **Epiglottic Movement** | | |
| (0) Complete | 10 (59%) | 10 (19%) |
| 1. Partial | 6 (35%) | 18 (35%) |
| 1. Absent | 1 (6%) | 24 (46%) |
| **Tongue Base Retraction** | | |
| (0) No contrast between tongue base and posterior pharyngeal wall | 0 (0%) | 0 (0%) |
| 1. Trace contrast | 1 (6%) | 0 (0%) |
| 1. Narrow contrast | 16 (94%) | 32 (62%) |
| 1. Wide contrast^P^ | 0 (0%) | 18 (35%) |
| 1. No posterior movement^P^ | 0 (0%) | 2 (4%) |
| **Pharyngeal Stripping Wave** | | |
| (0) Complete | 13 (76%) | 14 (27%) |
| 1. Partial | 3 (18%) | 28 (54%) |
| 1. Absent^P^ | 1 (6%) | 10 (19%) |
| **Valleculae Residue** | | |
| (0) None | 1 (6%) | 0 (0%) |
| 1. Trace | 5 (29%) | 6 (12%) |
| 1. Collection | 11 (65%) | 28 (54%) |
| 1. Majority^P^ | 0 (0%) | 9 (17%) |
| 1. No Clearance^P^ | 0 (0%) | 9 (17%) |
| **Pyriform Residue** | | |
| (0) None | 3 (18%) | 2 (4%) |
| 1. Trace | 6 (35%) | 8 (15%) |
| 1. Collection | 8 (47%) | 25 (48%) |
| 1. Majority^P^ | 0 (0%) | 8 (15%) |
| 1. No Clearance^P^ | 0 (0%) | 9 (17%) |
| **Pharyngoesophageal Segment Opening** | | |
| (0) Complete | 10 (59%) | 12 (23%) |
| 1. Partial | 6 (35%) | 21 (40%) |
| 1. Minimal^P^ | 1 (6%) | 10 (19%) |
| 1. None^P^ | 0 (0%) | 9 (17%) |

^P^ Demarcation for integrity categorized as ‘profound impairment’

*N=51 for components in domains III and IV due to poor image quality for one infant
